# Electrically switchable continuous phase liquid crystal Fresnel zone plate

**DOI:** 10.1038/s41377-026-02251-3

**Published:** 2026-04-17

**Authors:** Zhiyu Xu, Camron Nourshargh, Tianxin Wang, Alec Xu, Nathan Spiller, Urban Mur, Martin J. Booth, Steve J. Elston, Stephen M. Morris

**Affiliations:** Department of Engineering Science, University of Oxford, Oxford, OX1 3PJ UK

**Keywords:** Liquid crystals, Optoelectronic devices and components

## Abstract

We present the design, fabrication, and characterization of continuous phase Fresnel zone plates (FZPs) using two-photon polymerization direct laser writing in a polymerizable nematic liquid crystal (LC) confined between glass substrates. Unlike conventional binary LC diffractive elements, our devices exhibit a smooth, continuous three-dimensional phase profile. Two devices were demonstrated with wrapped phase profiles of 2*π* and 4*π* radians, respectively. Polarized optical microscopy and digital holographic microscopy confirm that the polymerized regions follow the intended spatially varying phase distribution. Far field measurements show that the 2*π* rad FZP generates a strong focal spot at 0 Vpp and switches off at higher voltages. In contrast, the 4*π* rad FZP exhibits varifocal behavior, switching between two focal lengths: 24 mm at 0 Vpp and 48 mm at an intermediate voltage of 2.1 Vpp. At higher voltages, the focus disappears entirely. Compared to a binary FZP of equal size and focal length, the continuous phase design nearly doubles the focusing efficiency and enables switchable, compact, vari-focal, and energy-efficient optical components. This approach offers new opportunities for advanced applications such as augmented and virtual reality, adaptive optics, and other next-generation photonic systems.

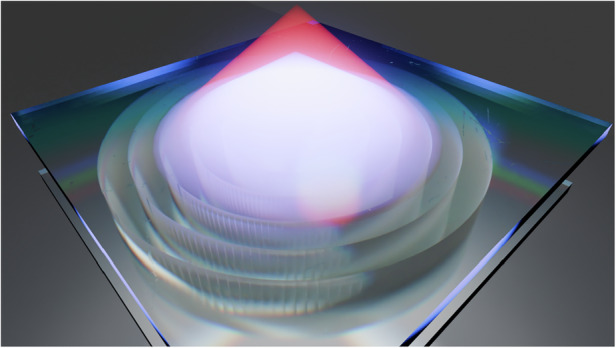

## Abstract (web summary)

This work demonstrates liquid crystal Fresnel zone plates with smooth 3D phase profiles, fabricated by direct laser writing, enabling dynamically switchable and reconfigurable optical focusing.

## Introduction

Liquid crystals (LCs) are a class of material positioned between conventional solids and liquids, exhibiting anisotropic optical properties that can be electrically, magnetically, or thermally controlled. Their unique electro-optic behavior arises from the molecular ordering of rod-like or disc-like molecules, which maintain a long-range orientational arrangement while preserving fluidity^1^. Unlike isotropic liquids, LCs possess directional-dependent (birefringent) refractive indices. This intrinsic birefringence, coupled with relatively low power consumption for molecular reorientation, has made LCs foundational in the development of displays, sensors, and emerging adaptive optical devices^2–9^. Over the course of several decades, a substantial body of research has leveraged these properties for new photonics applications, exploiting their rapid switching speeds, tunable phase retardation, and capacity for compact integration with existing manufacturing processes^10–12^.

Motivations for adopting LC technology in augmented reality (AR) and virtual reality (VR) systems are multifold. AR/VR headsets demand lightweight and compact optical components with high optical efficiency to deliver immersive user experiences^5^. Traditional refractive optics, typically composed of glass or plastics, often introduce bulkiness and additional weight that can be impractical for long-term or mobile applications^13^. Furthermore, as the resolution requirements for AR/VR displays soar, there is a growing need for active optical elements that can accommodate sophisticated wavefront shaping, image correction, and dynamic focusing. LCs, with their electrically controllable birefringence, promise these capabilities in a form factor well suited to miniaturization and integration. Their relatively low drive voltages, coupled with device architectures that can be engineered at micron scales, make them prime candidates for next-generation AR/VR optical modules such as adaptive lenses, switchable phase modulators, and dynamic diffraction gratings^14–16^.

Efforts to develop reconfigurable optical elements for compact light focusing have often turned to wavefront modulators of the form of deformable mirrors (DMs) and spatial light modulators (SLMs). While DMs rely on mechanical or electrostatic actuators to warp a reflective surface—enabling wavefront control in applications such as telescopes or high-power lasers, their moving parts introduce considerable fabrication complexity, cost, and reliability concerns. SLMs, on the other hand, can achieve dynamic focusing or phase modulation via arrays of individually addressable LC pixels. However, the resulting pixelation tends to produce diffraction artifacts (“screen door” effects), and the high voltages or intricate electrode designs often required can complicate integration into lightweight, power-efficient, and affordable AR/VR headsets. Consequently, simpler, more compact, and lower-power approaches are urgently needed to replace bulky conventional refractive optics.

An enormous amount of effort has already been devoted to developing LC-based optical elements for AR/VR devices. For instance, LC holographic optical elements (LCHOEs) that employ the use of photoalignment have been investigated. Due to their sensitivity to the input polarization state, combined with the intrinsic polarization control offered by the LC, such elements can be used to modulate the wavefront^17^. To date, LCHOEs have been widely applied to AR/VR for different functionalities, including pupil steering, off-axis lens arrays, diffractive waveguides, and varifocal lenses^6^.

Photoalignment-based LC diffractive optics/Pancharatnam-Berry (PB) geometric phase devices, which have been extensively explored, often involve interference exposure or direct-writing of the alignment layer, which enables both periodic and arbitrary geometric phase structures to be realized^18^. These approaches, however, fundamentally rely on a dedicated photoalignment layer, typically azobenzene-based materials such as SD1^19^. Importantly, photoalignment techniques intrinsically produce passive, polarization-selective geometric-phase/PB elements, where “switching” arises only from changing the incident polarization rather than from an active ON/OFF modulation of the phase profile itself. Moreover, the alignment state remains reversible, sensitive to moisture and oxygen, and often requires additional protective coatings. In practice, this multi-step alignment–coating–curing workflow can increase fabrication complexity and constrain device uniformity.

Separately, the use of holo imprinting has been shown to be an innovative approach to producing diffractive optic elements. Rather than relying on two-beam inference in free space, a master hologram template is used to project the information onto another substrate, which is potentially desirable for mass production and large-scale fabrication^20^. However, this approach is typically limited to reproducing in-plane optic axis distributions and therefore exclusively generates geometric-phase devices. Such elements are intrinsically spin dependent, producing a ±*Φ* conjugate pair of wavefronts for opposite circular polarizations. Furthermore, geometric phase devices replicated by holo imprinting are typically passive, and their phase function cannot be electrically suppressed; their apparent switching relies only on changing input polarization.

Greyscale lithography has also been employed to fabricate diffractive lenses, such as the large aperture photoresist devices reported by Aguiam et al. (10 × 10 mm^2^, NA = 0.25) that were fabricated using direct write greyscale lithography with multilevel relief defined by a discrete mask strategy^21^. The approach reported therein relies on calibrated dose to depth mapping and a layered/quantized surface relief profile (e.g., a 40-layer diffractive mask in their demonstration), which is highly effective for producing large area static diffractive optics. However, because the phase profile is ultimately implemented through a finite number of height levels and is fixed after fabrication, the resulting phase modulation is inherently discretized rather than continuously reconfigurable, and the optics are not electrically switchable. Overall, LC diffractive optical elements show promise in compact optical systems due to their ultrathin form factor, nearly 100% of theoretical efficiency, strong polarization selectivity, and switching ability^22,23^.

A significant challenge that remains lies in creating continuous or near continuous phase elements that circumvent SLM pixelation while retaining the switchability and low power consumption of LC devices. Researchers have begun to explore fabrication methods that harness the fluidity and photo-polymerizability of certain LC mixtures, which can be oriented under external fields and then *locked* into place upon curing. Two-photon polymerization direct laser writing (TPP-DLW) has emerged as a powerful tool in this context, thanks to its ability to create precise three-dimensional polymer networks at sub-micron resolution. By focusing a femtosecond laser into the LC mixture, TPP-DLW locally polymerizes targeted regions, thereby freezing the director orientation into a continuous phase distribution. In this way, the director inside the polymerized regions remained perpendicular after the removal of the electric field due to the polymer locking-in process^24^. This approach can yield smooth, passive, or actively reconfigurable devices with minimal scattering losses, which offer an essential leap towards truly compact, flat optical components that can be tuned/reconfigured using applied voltages that would be suitable for AR/VR and other advanced photonic systems.

Against this backdrop, Fresnel zone plates (FZPs) have gained renewed interest as thin, lightweight diffractive optical elements capable of focusing or imaging^14,25^. Conventionally, FZPs employ concentric rings with alternating transparent and opaque annuli or distinct phase steps to achieve focusing. In a two-dimensional (2D) binary arrangement, abrupt phase changes often yield multiple diffraction orders, thereby reducing the total efficiency in the principal order. For high-fidelity AR/VR displays, diffraction efficiency is paramount, as inefficient devices can lead to increased power consumption and suboptimal image brightness. The advantage of a continuous-phase FZP lies in its potential to minimize unwanted diffraction orders by smoothly varying the phase between zones, thus concentrating light more effectively into the primary focus. Additionally, switchable continuous-phase FZPs may find applications in other photonic systems requiring lightweight, compact focusing elements with minimal diffraction side lobes^14^. For example, compact imaging systems, beam-steering devices, and dynamic beam shapers in telecommunications could all benefit from a robust, reconfigurable diffractive optical element^3,26,27^.

**Fig. 1:**
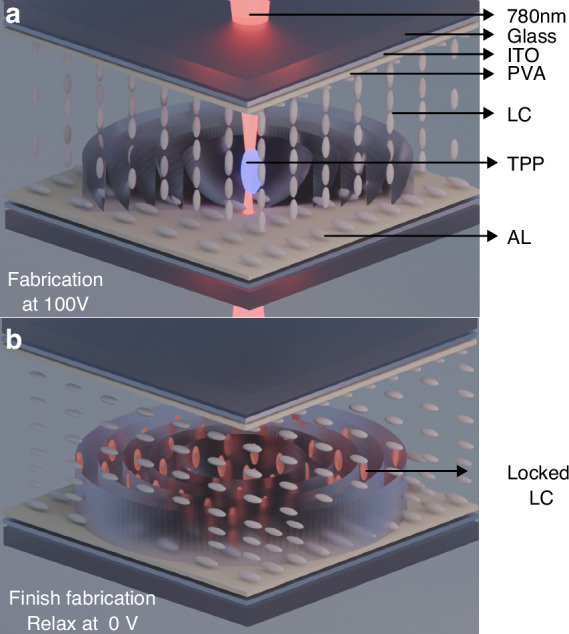
Fabricating a Fresnel Zone Plate (FZP) in a polymerizable liquid crystal (LC). **a** Illustration of fabricating continuous phase FZP using two-photon polymerization direct laser writing (TPP-DLW) in a polymerizable LC cell. The TPP-DLW locks the liquid crystal (LC) director by triggering two-photon polymerization inside the LC layer to form a rigid polymer network. The fabrication process is usually performed under a high voltage applied to the LC, resulting in a homeotropic alignment. **b** Illustration of a fabricated continuous phase FZP without an external electric field applied and the LC regions locked in a homeotropic alignment by the localized polymer network

In this work, we report a novel switchable continuous phase FZP fabricated using a polymerizable LC mixture inside a glass cell fabricated by TPP-DLW (Fig. 1). Our approach combines the benefits of a functional LC-based optical element (i.e., low power operation, tunable birefringence, and compact form factor) with efficiency gains derived from a continuous rather than binary phase structure. By employing a carefully engineered LC formulation and TPP protocol, we establish a stable baseline orientation that defines the continuous phase distribution required for focusing. In operation, applying an external voltage modifies the local director orientation within the LC matrix, allowing for a real-time switch of the focal length between two focal planes. Such reconfigurability could be highly attractive for immersive display headsets, where dynamic optical power might compensate for user-specific vision correction or enable real-time focal plane adjustments that enhance comfort and immersion. Furthermore, by mitigating the pixelation inherent to SLM-based approaches, our device can maintain higher optical efficiency and yield clearer, more precise wavefront control crucial for advanced display engineering.

The approach presented here introduces a fundamentally different mechanism that involves a direct 3D refractive index sculpting of a polymerizable LC medium using TPP-DLW, which avoids the need for photoalignment materials entirely and enables a genuinely switchable continuous phase FZP whose operational state is defined electrically rather than by the illumination polarization. Furthermore, our TPP-DLW approach directly sculpts the dynamic phase optical path length, enabling a scalar continuous phase profile that does not split into conjugate orders. This class of continuous phase device cannot be readily produced by holo imprinting techniques, which are restricted to polarization-selective geometric phase devices. Second, by embedding the printed polymer structure directly inside a voltage-tunable LC layer, we achieve true electrical ON/OFF switching of the focusing functionality. The TPP-DLW in polymerizable LC strategy enables electrically switchable phase state control within the same continuous phase design framework, allowing dual-state (discrete) varifocal operation and direct experimental validation of phase evolution under applied electric fields. This capability offers a distinct advantage for tunable diffractive optics, where dynamic phase control is as critical as static profile fidelity, and provides a new operational degree of freedom typically absent in alternative fabrication modalities.

## Results

### Design and fabrication

**Fig. 2:**
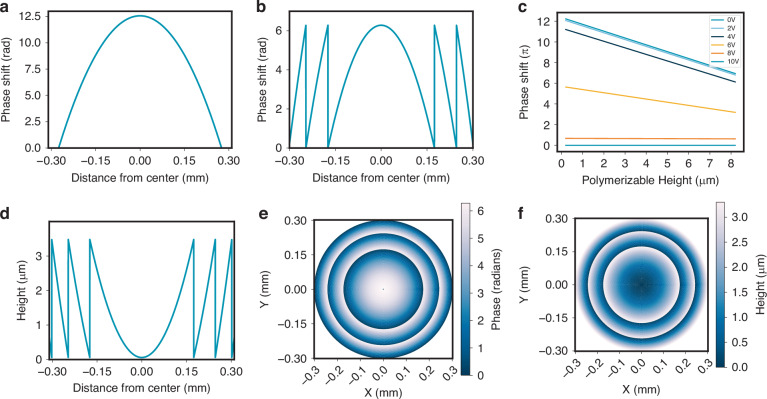
Parameters used for the design and fabrication of a laser-written Fresnel zone plate (FZP). **a** Unwrapped phase profile for the designed FZP. **b** Wrapped phase profile showing periodic discontinuities within the 2*π* rad range. **c** Correlation of $\Delta \phi$ and polymerization height within the LC layer using the Euler–Lagrange relaxation method (see “Materials and methods”). **d** The reconstructed height profile for the FZP calculated from the optimized polymerization parameters. **e** 2D simulation of the spatial dependence of the phase profile of the continuous-phase FZP and (**f**) the corresponding polymerization height profile across the *x*–*y* plane for the continuous FZP

In this study, we first fabricated a 2*π* rad wrapped continuous FZP in a 20 µm air gap anti-parallel rubbed LC glass cell filled with a polymerizable LC mixture prepared by 78 wt.% of the nematic LC mixture E7 (Synthon Chemicals Ltd.), 20 wt.% of the reactive mesogen RM257 (Synthon Chemicals Ltd), and 1 wt.% of the Photoinitiator IR819 (see “Materials and methods”). The continuous FZP was designed to have a diameter of 600 µm, with a maximum polymerized height of approximately 4 µm. The lens was designed to focus incident light of 633 nm with a focal length of 30 mm. A phase profile of the corresponding lens was generated, as shown in Fig. 2a. The maximum phase in this case reaches 4*π* before wrapping, which is redundant for focusing optics, and so a wrapping by 0–2*π* rad was carried out. This wrapped profile maintains the correct phase progression for focusing while ensuring practical implementation in a physical LC device. By applying the phase-height conversion function (see “Materials and methods”), the shape of the wrapped FZP could be determined as shown in Fig. 2b.

The reactive mesogen concentration in the polymerizable LC mixture is also vital to achieve a smooth and accurate phase profile. Empirically, 20 wt.% or higher concentration of RM257 was found to result in a stable and rigid polymer framework, which helps the formation of smooth phase profiles. While for lower reactive mesogen concentrations, the polymer network formed by the TPP-DLW was not found to be sufficiently robust and tended to lead to inhomogeneities in the fabricated structure and resulting phase profile.

Our TPP-DLW method offers a very small and precise voxel for polymerization with a lateral size of approximately 1 μm diameter in the *xy*-plane and an axial extent of about 7 μm along the *z*-axis. Subsequent diffusion notwithstanding, the shape and the size of the voxel determines the polymerization region because only inside the voxel is the incident light intensity high enough that two photons can combine their energy to trigger TPP, eventually locking the director in place^28^. Furthermore, the fabrication process was initiated intentionally at the lower surface of the LC cell, and the polymer formed by chain reaction then reaches the upper glass surface inside the LC cell if the polymerization height is beyond 8 µm, verified after several practical attempts. This would impose a homeotropic alignment throughout the entire thickness of the LC layer along the *z*-axis and would lead to failure to control the director profile in that axis. Thus, empirically, we limited the possible writing height to around 7 µm to prevent such failure. For this 2*π* rad wrapped FZP, the maximum height was designed to be around 3.5 µm, which was within the polymerizable height inside the 20 μm-thick LC layer. The fabrication time for a 600 µm diameter continuous phase FZP, with optimization of the writing resolution, writing speed and power, could be achieved within 30 min. For a 1.2 mm diameter continuous phase FZP, the writing time could be optimized to be less than 3 h.

As the nematic LC is also optically anisotropic, the electric field was used to reorient the LC director angle, *θ*, which is related to the effective refractive index according to

1 $${n}_{\mathrm{eff}}\left(\theta \right)=\sqrt{\frac{{n}_{e}^{2}{n}_{o}^{2}}{{n}_{o}^{2}{\cos }^{2}\left(\theta \right)+{n}_{e}^{2}{\sin }^{2}\left(\theta \right)}}$$

where ${n}_{e}$ is the extraordinary refractive index and ${n}_{o}$ is the ordinary refractive index; $\theta$ is the director angle with respect to the $z$-axis. During fabrication, a voltage of 100 Vpp was applied, ensuring that the director aligned perpendicular to the alignment layer, followed by exposure to the laser. Practically, fabricating the continuous FZP pattern both with and without the voltages applied has been carried out. When the phase profile pattern was written at 100 Vpp, the LC device functioned as a continuous FZP at 0 Vpp as the phase retardation resulted from the contrast between the polymerized regions and the nonpolymerized region, for which light passing through experienced *n*_*e*_. In contrast, when the phase profile pattern was written at 0 Vpp, the lens functioned at 100 Vpp as the phase retardation was provided by the polymerized part in the LC in which the incident light experienced *n*_*e*_. While both approaches have the potential for focusing light, fabricating the pattern at 0 Vpp revealed some drawbacks.

**Fig. 3:**
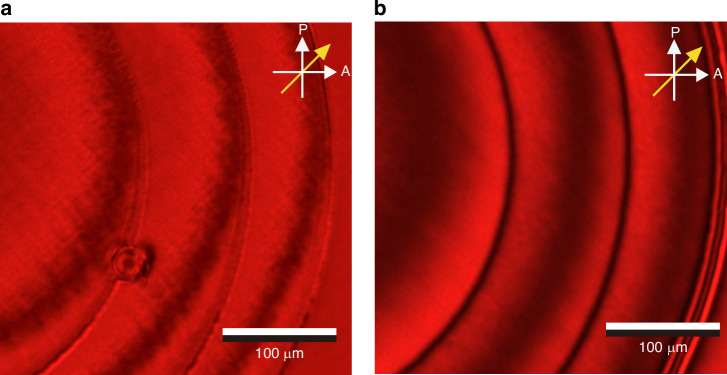
Fabricating the Fresnel Zone Plate at different voltage conditions. Representative polarizing optical microscope (POM) images (with a red 660 nm to 694 nm bandpass filter, inserted after the halogen bulb of the microscope) of continuous phase FZPs fabricated at either a write voltage of (**a**) *V* = 0 Vpp or (**b**) at *V* = 100 Vpp. The single-headed white arrows indicate the orientations of the polarizer (P) and analyzer (A), while the single-headed yellow arrows represent the rubbing directions of the alignment layers

Firstly, for an FZP written at 0 Vpp, the optical element only functions as a lens when certain voltages are applied, which means it cannot act as an optical element at 0 Vpp, and it must therefore be switched ON to focus light. In doing so, power is consumed whenever the device is driven into its on state. Also, the pattern fabricated at 0 Vpp did not appear as uniform compared to the one fabricated at 100 Vpp. As shown in Fig. 3, the 0 Vpp written polymer exhibited a less well-defined polymer structure, as evidenced by the corresponding polarizing optical microscope (POM) image, while the 100 Vpp written polymer network showed a much smoother profile (see Fig. 3b). The difference in behavior for the two different fabrication conditions may be due to thermal fluctuations, wherein random fluctuations in the LC director arise in the absence of an applied voltage confinement resulting in a less well-defined polymer network. Triggering photo-polymerization at large voltage amplitudes appears to resolve this problem. Because the phase profile is related to the configuration of the spatially variant polymer network, which locks in the director orientation, maintaining a smooth and precise director orientation in 3D during fabrication is essential for the resulting performance of the FZP.

In addition, the 20 wt.% RM257 concentration was selected to balance optical phase modulation and structural robustness. Although this loading is higher than in many traditional polymer-stabilized LC systems, reducing RM257 to a concentration of 15 wt.% produced visibly blurred structures as can be seen from the polarizing optical microscope (POM) image (Supplementary Information Fig. S1), indicating insufficient network formation and likely contributing to nonideal phase profiles. Increasing RM257 can modify the prepolymer effective refractive index/birefringence and therefore influence phase delay; while our simulations did not explicitly incorporate a detailed concentration-dependent optical model, experimental results confirm that the chosen mixture provides adequate Δ*n*_eff_ for the 4*π* rad wrapped FZP while ensuring mechanical stability and preventing voltage-induced deformation of the written polymer network. The 1 wt.% photoinitiator concentration was chosen to ensure reliable polymerization under our writing conditions; within the studied voltage range, we did not observe degraded electro-optic tunability in the nonpolymerized regions. E7 was selected as a well-characterized nematic LC host that provides stable, reproducible baseline properties for modeling and comparison; index matching with polymerized RM257 was not the primary design criterion but likely aids alignment stability. Using a higher birefringence could potentially expand the phase modulation range and relax the required RM257 fraction, though at the cost of increased sensitivity to anchoring and potentially altered switching dynamics.

To assess the optical impact of polymerization within the LC mixture, transmission measurements were carried out (see Supplementary Information Fig. S2). A small decrease in transmission of roughly 5% was observed after curing, consistent with the formation of a weak polymer network that introduces minor refractive index fluctuations and correspondingly slight scattering. Although this confirms that polymerization does alter the optical environment slightly, the magnitude of the change remains modest and does not noticeably affect device performance under the operating conditions used in this study. With further optimization of mixture composition and photo-polymerization parameters, the residual scattering could be reduced even further.

### Characterization of the 2*π* wrapped continuous phase FZP

**Fig. 4:**
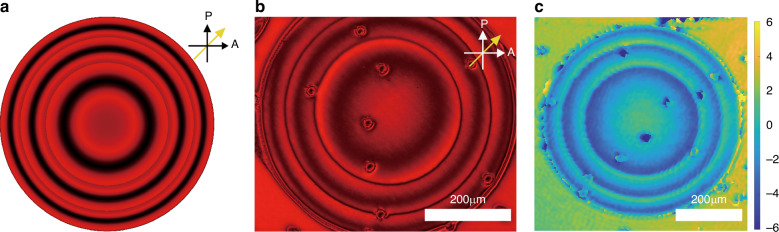
A 2π wrapped continuous phase Fresnel Zone Plate. **a** Simulated POM image of a wrapped continuous nematic LC FZP. **b** POM image of the fabricated continuous phase FZP when viewed with crossed polarizers obtained from experiments. The diameter of the FZP is 600 µm, and the focal length is *f* = 30 mm for a 20 µm thickness LC cell. The black and white arrows in (**a**, **b**) represent the orientations of the polarizer (P) and analyzer (A), respectively. The yellow single-headed arrow shows the orientation of the rubbing direction of the nematic LC device. The round particles in the fabricated patterns are spacer beads, which hold the thickness of the cell. **c** The phase profile of the FZP extracted from the results obtained on a digital holographic microscope

The continuous FZP fabricated at 100 Vpp was further imaged on a POM, whereby the rubbing direction was aligned at 45° to the transmission axes of the polarizer/analyzer pair. The FZP was also simulated using the same set of parameters (*λ, r, f*) as that employed in the experiments. In both the simulated POM images as well as those obtained in experiments (Fig. 4), the thin black ring shown represents the discontinuity where Δ*ϕ* equals *π* rad (i.e., out of phase). Inside each discontinuity, from the inner to the outer, the intensity changes from a bright to dark state before returning to a bright state, which indicates that Δ*ϕ* changed from 0 (constructive interference) to *π* rad (destructive interference) to 2*π* rad (constructive interference).

The simulations (Fig. 4a) match well with the actual image captured by the POM in the experiments (Fig. 4b) and provide a preliminary confirmation that the wrapped phase profile of the lens agreed well with the design. To further confirm the phase profile quantitatively, the fabricated continuous FZP was then examined using a digital holographic microscope, which is able to extract the phase information of microscopic objects. As shown in Fig. 4c, the phase at the center of the FZP takes the highest value, which decreases upon moving away from the center to the edge of each ring, indicating that Δφ decreases and confirming the desired curvature of the lens profile.

### Focusing performance and binary comparison of the 2*π* wrapped FZP

After validating the shape and phase curvature of the 2*π* rad wrapped FZP, characterization of the focusing effect of the lens was carried out using the experimental configuration (see Supplementary Information Fig. S3). For a nematic LC in a glass cell with anti-parallel rubbed alignment layers, the macroscopic optic axis will tend to align with the rubbing direction. This results in polarization sensitivity to the incident light. Thus, only when the polarization of the incident light is parallel to the rubbing direction can the light be manipulated overall by the LC FZP.

In the transmission experiment, a continuous-wave He–Ne laser emitting at *λ* = 632.8 nm was used to characterize the focusing properties of the FZP, which was chosen as it is a monochromatic light source with a well-defined linear polarization. The orientation of the linear polarization was subsequently rotated to be parallel with the rubbing direction by rotating the combination of the linear polarizer (LP) and HWP.

**Fig. 5:**
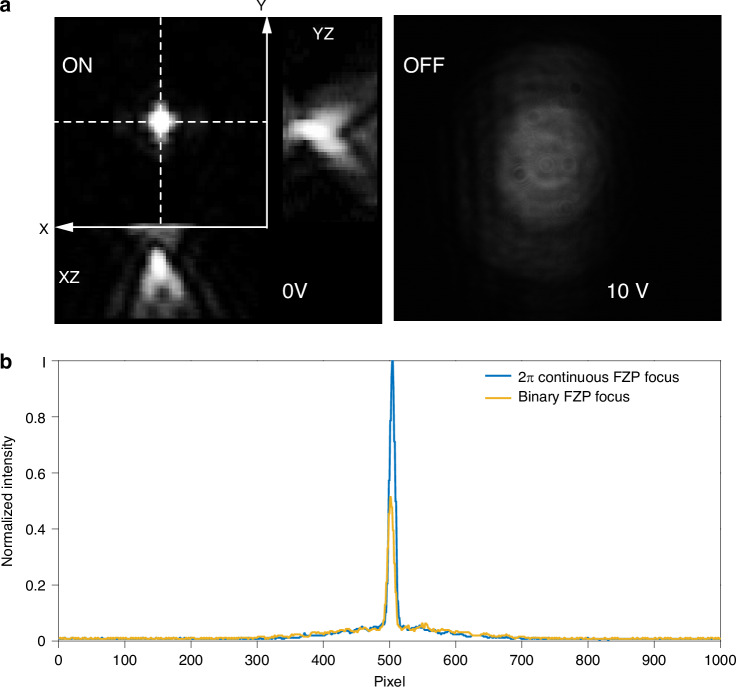
Focusing characteristics of binary and continuous 2π rad wrapped Fresnel Zone Plates. **a** Images of the focal plane when the laser-written FZP was illuminated with a 633 nm He–Ne laser. The left image shows the appearance of a focal spot in the *xy*-plane and corresponding images for the *xz*- and *yz*-planes when no voltage is applied (in this case, the FZP is effectively active). The right image shows the disappearance of the focal spot when a voltage of 10 Vpp is applied, thereby deactivating the FZP. **b** Normalized intensity at the focal plane for the laser-written continuous phase FZP and a binary FZP (see “Materials and methods”) designed to have the same focal length and device diameter

The actual focusing behavior observed is shown in Fig. 5a. When the voltage applied to the LC FZP was 10 Vpp, the far-field image was found to be blurry and dark. This indicates that although the laser polarization state was set to be parallel to the LC cell rubbing direction, the laser was not manipulated by the continuous phase FZP. Alternatively, when the signal generator was switched off, a bright and sharp focus was captured at the designed focal plane.

The observed phenomenon aligns well with our expectations that a wrapped phase profile for the FZP was created by forming a polymer network at well-defined locations within the LC layer to lock the director into the predetermined spatially varying director profile. Furthermore, one of the most important benefits of a continuous phase FZP compared with a binary FZP is the efficiency. Traditionally, binary FZPs have been successfully fabricated using many different approaches inside LC cells, such as using mask projection and patterned electrodes^14,29^. However, such approaches might also lead to limitations in terms of the achievable resolution due to the precision of the fabrication process. More importantly, binary FZPs do have a principal focus, but power is distributed amongst multiple diffraction orders, reducing efficiency in the desired orders. This results in a loss of intensity in the focal spot, which leads to low efficiency. Comparatively, the continuous phase FZP focuses all the incident light into one order, which, in theory, ensures 100% efficiency.

In this experiment, a binary FZP designed for the same incident wavelength, the same dimensions, and with the same focal length was also fabricated for the purposes of comparison. The binary and continuous phase FZPs were both fabricated in the same LC cell at different locations for consistency. Figure 5b illustrates that the normalized intensity at the focus of the continuous phase FZP is almost double that of the binary FZP, with a measured ratio of approximately 196%. This intensity comparison confirms the expected improvement in light efficiency as well as the benefits of developing continuous phase FZPs for more advanced near-eye optical applications. Such observations unequivocally confirm that the continuous phase FZP presented herein is capable of efficiently focusing collimated light, enabling precise spatial convergence and controlled beam shaping.

### Design and phase characterization of a voltage switchable 4*π* wrapped FZP

Up to this point, we have demonstrated a highly optically efficient continuous 2*π* rad wrapped FZP that is switchable. The next step was to demonstrate whether a continuous FZP could be developed that enabled a change between two discrete focal lengths, a so-called varifocal FZP. Here, varifocal behavior was achieved by wrapping the lens phase profile so as to be 4*π* rad instead of 2*π* rad.

**Fig. 6:**
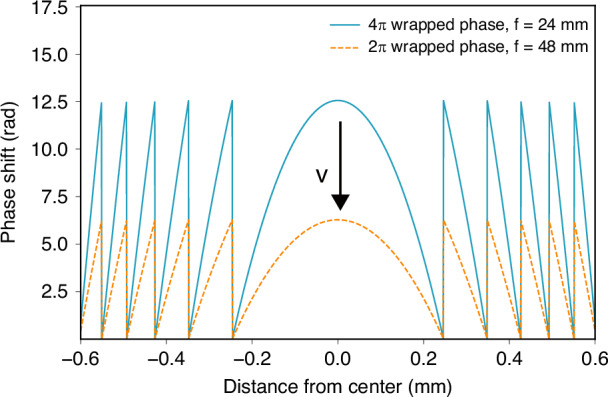
Comparison (simulations) of 2π and 4π rad wrapped continuous phase Fresnel Zone Plates. Simulated phase profile of a 4*π* rad wrapped FZP with a focal length of *f* = 24 mm (solid blue line) and a 2*π* rad wrapped FZP with a focal length of *f* = 48 mm (dashed red line)

The focal length is determined by the curvature of the lens profile, as for the continuous phase FZP described above. Through a novel design, a single FZP in a polymerizable LC can actually achieve two different focal lengths with the extra ability to switch between them. As an example, shown in Fig. 6, a 4*π* rad wrapped FZP with a designed focal length of *f* = 24 mm is found to exhibit ten periodic phase discontinuities within a 1.2 mm diameter range. Comparatively, a 2*π* rad wrapped FZP with *f* = 48 mm can also be made to exhibit ten phase discontinuities within the same 1.2 mm diameter range at the exact same geometric positions. This matching in geometric position of the phase discontinuities guarantees that the phase profiles remain continuous when unwrapping; otherwise, the phase profile breaks apart, and it is no longer a continuous lens.

Building upon this concept, when a 4*π* rad wrapped phase profile is generated inside the polymerizable LC mixture, the phase profile can be controlled using an applied electric field in such a way that no longer results in a straightforward switching ON and OFF of the FZP. Instead, an intermediate voltage can be found such that the focal length can be tuned between two focal planes. To explain the reason for this, consider the planar-aligned nonpolymerized LC regions inside the cell in the absence of an applied voltage. Assuming the polarization of incident light is parallel to the optic axis in these regions, the light experiences maximum retardation (*n*_*e*_ - *n*_*o*_) at 0 V. By gradually increasing the applied voltage, the LC will tend to align with the external electric field, so that the light experiences smaller and smaller retardation as the voltage increases. For a 4*π* rad wrapped FZP, when applying higher voltages, the phase profile will gradually decrease until it matches the phase profile of a 2*π* rad wrapped FZP, but with double the focal length of that for the 4*π* rad configuration. In this case, the LC device will only be able to act as a lens if both phase profiles have the same discontinuity profile.

It is important to emphasize that this design principle does not imply a continuously tunable focal length in the strict sense. Rather, the device is engineered to support two discrete, optically functional focal planes: one associated with the full 4*π* modulation at low voltage and a second associated with the reduced 2*π* equivalent modulation at a higher, intermediate voltage. While the phase amplitude evolves continuously with the applied electric field, the requirement of matched discontinuity profiles means that high-quality imaging performance is expected primarily near these two designed operating points. Outside these voltage conditions, the phase profile may deviate from an ideal lens-like form, leading to reduced focusing efficiency and increased aberration. Consequently, the device should be interpreted as a bi-stable (or dual-state) varifocal element that switches between two well-defined focal planes, rather than a broadly continuous, analog tunable lens across the full voltage range.

**Fig. 7:**
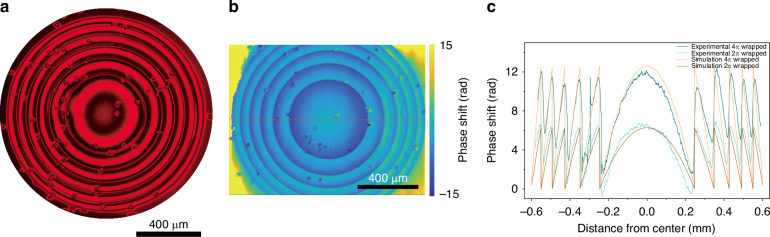
A 4π rad continuous phase Fresnel Zone Plate. **a** A POM image of the laser-written wrapped 4*π* rad continuous phase FZP with a diameter of 1.2 mm and a focal length of *f* = 24 mm. **b** The corresponding phase profile of the FZP extracted using a digital holographic microscope (DHM). **c** A cross-section of the phase profile of the 4*π* rad wrapped continuous phase FZPs along the red dashed line in (**b**). Results obtained from both simulations and experiments for the 4*π* and 2*π* rad wrapped continuous phase cases are presented

To demonstrate this bi-stable (or dual-state) varifocal functionality, a 1.2 mm diameter continuous phase FZP wrapped into 4*π* rad was fabricated with a focal length *f* = 24 mm, as shown in Fig. 7a. The experimentally obtained POM image shows a smooth, continuous phase FZP with relatively sharp discontinuities between each 2*π* rad wrapping. By carefully examining the central circle, the color change moving away from the center undergoes a red–black–red–black transition, where the black pattern appears due to Δ*ϕ* = *π*, indicating destructive interference. In every 2*π* of phase, there is a transition in color from bright to dark. In this case, two periods of bright-to-dark transitions appear, which implies that a total phase wrap of 4*π* has been achieved.

Digital holographic microscopy was then used to scan the continuous phase FZP for a quantitative measure of the phase profile, as shown in Fig. 7b. It can be seen that there is a clear jump in the phase for each discontinuity. Noticeably, the right part of the FZP has a smaller absolute phase value than the left part. There are two possible reasons for this: firstly, the wavefront of the incident light from the digital holographic microscope may not have been perfectly planar, so that the phase at different regions is shifted. Secondly, a small tilt of the fabrication surface during the laser writing process may have caused a resulting tilt in the final FZP phase profile.

In Fig. 7c, the cross-sections of the reconstructed phase profiles of the continuous-phase FZP at 0 Vpp and 2.1 Vpp are shown. At 0 Vpp, the measured 4*π*-wrapped phase distribution closely follows the designed profile, with a maximum phase modulation of 11.5 rad, corresponding to approximately 4*π*. When the applied voltage is increased to 2.1 Vpp, the maximum phase excursion is reduced from 4*π* to 2*π*, while the locations of the phase discontinuities remain essentially unchanged. This voltage-dependent scaling of the phase amplitude, together with the preserved zone structure, confirms that the applied voltage effectively tunes the phase profile of the FZP and, hence, its effective focal length. The phase profile extracted from digital holographic microscope suggests that the apparent phase modulation is reduced in the outer zones: while the peak phase can remain close to the designed maximum, the minima are elevated relative to the central region. This trend is most consistent with a measurement-limited artifact rather than a genuine change in device response. As the radius increases, the FZP zone width decreases, and the steep phase transitions at the zone boundaries approach the effective lateral resolution of the digital holographic microscope.

The recovered phase, therefore, represents a spatially averaged version of the true profile, which blurred abrupt jumps, preferentially “fills in” narrow valleys, and yields a higher baseline and smaller apparent phase swing, with increased uncertainty in both boundary position and jump magnitude. Reduced fringe contrast at large radii, together with more fragile phase unwrapping and background subtraction, can further bias the retrieved phase toward smoother, higher minimum profiles. Apart from the resolution limit of the digital holographic microscope, the slight residual discrepancies between the simulated and experimentally retrieved phase profiles are likely due to a small tilt of the LC cell during fabrication and/or a weakly non-uniform wavefront in the digital holographic microscope measurements. Future improvements could involve improved tilt control both before the laser writing process as well as during the process, which would ensure an even higher quality of the phase profile. Also, higher numerical aperture microscope objectives could be explored to generate a smaller voxel size, thus enabling higher resolution writing, which might also help to further improve the phase profile.

### Voltage-controlled focal plane switching and propagation behavior

**Fig. 8:**
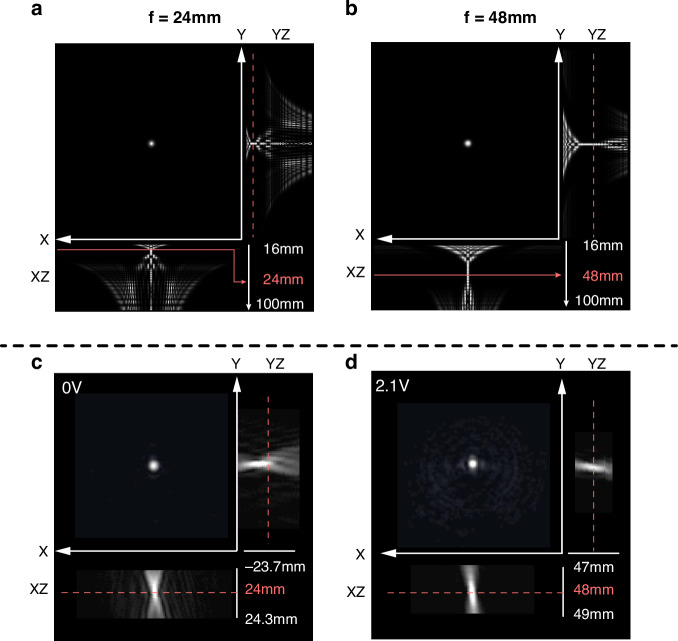
Focusing characteristics (simulations and experiments) for 4π rad wrapped continuous phase Fresnel Zone Plates. Simulated focal spots and corresponding propagation in the *XZ*- and *YZ*-planes for (**a**) a 4*π* rad wrapped FZP and (**b**) a 4*π* rad wrapped FZP with compressed maximum phase, both at the designed focal length of *f* = 24 mm. **c**, **d** Experimental results obtained for the configurations presented in (**a**, **b**), respectively

Far-field focusing measurements were then carried out using the same optical arrangement (See Supplementary Information Fig. S3). A comparable simulation of the propagation of light through the 4*π* rad FZP was also performed and is shown in Fig. 8a, b. This simulation was based on the ideal wrapped lens phase profile without considering the actual behavior of the LC due to the presence of the polymerized LC network within the glass cell. The unconstrained nonpolymerized LC regions adjacent to the homeotropically-aligned polymerized LC regions will gradually relax to a planar alignment, resulting in a transition region where the effective refractive index will vary smoothly, especially at the discontinuities in the FZP. This profile will then cause a blurring of the edge of the discontinuity, which might negatively influence the overall phase profile. However, for our simulations, we took the ideal phase profiles with which to compare the experimental results. In spite of this oversimplification, the simulations were found to be in good agreement with experiments.

In Fig. 8a, c, both images (simulations and experiment) show a sharp focus spot at *f* = 24 mm. In the *XZ*- and *YZ*-planes, along the propagation direction, the beam converges towards its minimum radius *ω*_0_ at the focal plane and subsequently diverges, forming a characteristic hourglass-shaped waist profile governed by Gaussian beam propagation. Similarly, when the LC cell was driven at 2.1 Vpp, another focus at *f* = 48 mm was observed, which is exactly double the focal length in accordance with simulations. Closer inspection reveals that the focus is less than ideal, with some intensity of light appearing around the focus. This could be due to imperfections in the overall phase profiles at 2.1 Vpp because of the fabrication imperfections described previously. In this case, due to the errors in the fabrication, some of the wrapped phase regions may not result in the formation of a smooth curvature of the phase, causing some diffraction of the light to occur.

At 0 Vpp, the FZP produces a relatively short focal region, while at 2.1 Vpp, the focal region is significantly longer; this arises because the longer focal length reduces the beam’s convergence angle, thereby increasing the Rayleigh range and extending the waist along the propagation axis. The spatial quality of the reconfigurable focus was quantified by analyzing the focal-plane intensity distributions at *f* = 24 mm (0 Vpp) and *f* = 48 mm (2.1 Vpp) (see Supplementary Information Fig. S4). For each voltage, the charge-coupled device (CCD) image of the focus was azimuthally averaged to obtain a one-dimensional radial intensity profile, which was then normalized to its peak value (solid lines), while the shaded region indicates the standard deviation over different azimuthal angles. In both cases, the profiles exhibit a single, narrow central maximum with negligible background, indicating that the FZP produces a well-defined, high contrast focus with no significant broadening when the focal length is switched. The side lobes appear only as weak oscillations around the baseline and remain much lower in amplitude than the main peak, consistent with good suppression of higher-order diffraction and aberrations. The small standard deviation further confirms that the focal spot is nearly circularly symmetric. Overall, the matching of voltages and focal lengths demonstrates the ability of a 4*π* rad wrapped continuous phase FZP to switch the focal length by simply using an applied voltage without any additional mechanical movement of the device. Also, these spot line profile measurements demonstrate that the switchable FZP maintains a near-diffraction-limited focal spot and good side-lobe suppression at both *f* = 24 mm and *f* = 48 mm.

**Fig. 9:**
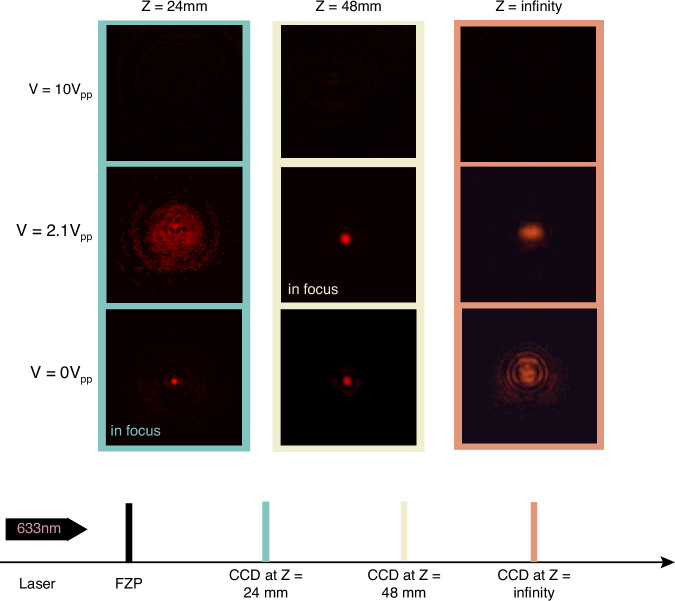
Focusing behavior of the laser-written wrapped 4π rad continuous phase Fresnel Zone Plate. Results are shown for a FZP with a diameter of 1.2 mm and a focal length of f = 24 mm at three different applied voltages and at three different propagation distances

Experimental characterization of the focusing for different applied voltages and propagation distances was carried out, and the results are shown in Fig. 9. All of the images were taken on a CCD camera that was set to the same exposure and gain. At 0 Vpp, where the designed 4*π* rad wrapped FZPs should focus to a distance of 24 mm, a sharp and bright focal spot was observed at this exact propagation distance. Then, when the CCD camera was placed at *Z* = 48 mm, the camera captured a larger and blurrier spot surrounded by darker rings. Theoretically, at *Z* = 48 mm, the FZP should not focus light, but instead the light should largely diverge because the lens profile was designed to focus the incident laser light at *Z* = 24 mm at 0 Vpp. In practice, however, a weak, blurry spot was detected, meaning that the actual phase profile at 0 Vpp also resulted in the manipulation of light at a distance of *Z* = 48 mm. This behavior might be caused by the phase profile being slightly less than the intended 4*π* rad wrapping, as indicated earlier in Fig. 7c, where the measured phase was found to be around 11 rad. This would lead to a slight mismatch of the reconstructed phase profile, resulting in a pillar-like binary phase FZP, which is also possibly the reason for the formation of a blurry spot at *Z* = 24 mm. When moving the CCD camera to a larger distance beyond *Z* = 48 mm, the laser light diverged, as expected.

At 2.1 Vpp, when the observation plane was placed at *Z* = 24 mm, the recorded intensity distribution appeared strongly defocused, whereas a well-defined, sharp focal spot was obtained at *Z* = 48 mm. In this condition, the focus was brighter, and the surrounding ring-like intensity present at 0 Vpp largely disappeared, indicating that the phase profile of the FZP at 2.1 Vpp is close to an ideal 2*π*-wrapped kinoform with a focal length of *f* = 48 mm. When the camera plane was moved toward the far-field (effectively *Z* → ∞), the transmitted beam was observed to diverge, as expected for a focusing element. At an applied voltage of 10 Vpp, no clear focusing behavior was observed at any of the three image planes, confirming that the lens profile is effectively switched OFF due to the nearly uniform homeotropic alignment of the LC director across the device.

**Fig. 10:**
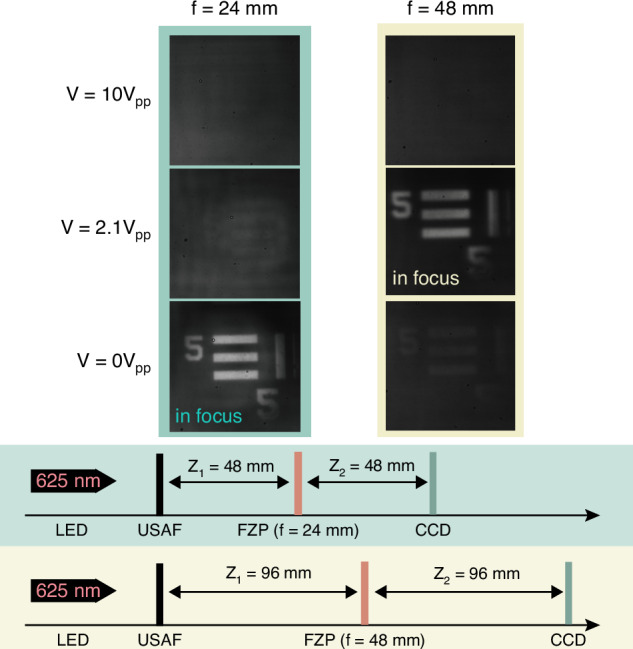
Image formation using the continuous phase FZP with varifocal behavior. The focal length of the FZP is switched between f = 24 mm and f = 48 mm by applying different voltages

To demonstrate the imaging capability of the device, a USAF 1951 target was used as the object. As shown in Fig. 10, an optical imaging system was assembled to measure the varifocal ability (see “Materials and methods”). First, *Z*_1_ and *Z*_2_ were set to be a total separation of 48 mm in order to test the imaging ability for the case when the FZP had a focal length of *f* = 24 mm, in accordance with the thin-lens equation

2 $$\frac{1}{f}=\frac{1}{{z}_{1}}+\frac{1}{{z}_{2}}$$

where f is the focal length of the thin lens, *Z*_1_ and *Z*_2_ are the object distance and image distance, respectively. This arrangement resulted in a 1:1 magnification. For *f* = 24 mm, the designed working voltage is 0 Vpp, and the image observed on the CCD showed a clear and sharp USAF target pattern, as expected. Then, when the voltage was switched to 2.1 Vpp, which was the design voltage for the case when *f* = 48 mm, the pattern disappeared. At 10 Vpp, the LC will tend to align homeotropically so that the lens profile is removed, resulting in no image being formed in this case. Similarly, when examining the imaging capability for *f* = 48 mm, *Z*_1_ and *Z*_2_ were set to 96 mm to achieve the same 1:1 magnification. At 0 Vpp, which is not the design voltage for the case when *f* = 48 mm, a very vague image was captured on the CCD, which might be caused by the phase profile imperfections that arise from the fabrication process, as mentioned earlier.

However, at 2.1 Vpp, a clear image is observed as this is the design voltage for the FZP to exhibit a focal length of *f* = 48 mm. It is noticeable that the resolution of the image at *f* = 48 mm is somewhat reduced compared with that observed at *f* = 24 mm. Since the two focal states share the same physical aperture, the numerical aperture scales as NA ≈ *R*/*f*. Therefore, increasing the focal length from *f* = 24 mm to *f* = 48 mm approximately halves the NA, and hence doubles the diffraction-limited spot size *δ* ≈ 0.61*λ*/NA. As a result, the *f* = 48 mm state exhibits a reduced lateral resolution relative to the *f* = 24 mm state. Collectively, the results presented here demonstrate that the 4*π* rad wrapped FZP can switch between two different focal lengths effectively.

It is important to note that the present device behaves as a discrete, order-switchable kinoform rather than as a continuously tunable lens. At 0 Vpp, the LC director is in its low electric field configuration, and the effective phase depth is close to 4*π* at the design wavelength, so that the continuous profile focuses to the shorter design focal length (*f* = 24 mm). When the voltage is increased to approximately 2.1–2.2 Vpp, the LC-induced retardation is reduced to around 2*π*, and the same geometric phase function operates as an effective first-order kinoform with a longer focal length of *f* = 48 mm. At higher voltages, the LC approaches a homeotropic-like state, and the effective birefringence is therefore strongly diminished. This results in the phase modulation being largely suppressed, leading to a non-focusing OFF state. Intermediate voltages between these operating points (voltages), in theory, should produce mixed-order responses, in which the optical power is distributed between multiple diffraction orders and the axial intensity profile exhibits broadened or partially overlapping foci rather than a single well-defined focal plane. Consequently, this implementation offers two robust voltage addressable focal states (*f* = 24 mm and *f* = 48 mm) and an electrically controlled OFF state, but does not realize a continuously varying focal length as a function of applied voltage.

Owing to the enlarged phase dynamic range associated with the 4*π* rad wrapped design, small deviations from the ideal height profile give rise to residual light leakage and slight focal broadening. In our devices, these imperfections are likely to arise from three main sources. First, a residual substrate tilt introduces an unintended linear phase ramp across the aperture; this can be mitigated by improved mechanical tip–tilt control during writing and by numerically pre-compensating the design with a corrective linear phase term. Second, LC relaxation and director non-uniformity lead to local birefringence variations, particularly at intermediate voltages; enhanced alignment control, more uniform spacer distribution, and optimized driving schemes can reduce this effect. Third, the finite voxel size and limited sampling of the outer Fresnel zones constrain the fidelity of the realized 4*π* profile. Higher NA objectives, finer writing grids, and wavefront-corrected exposure (e.g., SLM-based aberration compensation) offer clear routes to improving fabrication accuracy. Together, these refinements provide a practical pathway toward higher phase fidelity and near-diffraction-limited performance in future iterations of the device.

### Long-term electro-optic stability and response times

The long-term electro-optic stability and response time of voltage switchable devices are often critical for practical deployment. To assess the robustness of the proposed FZP under prolonged electrical driving, a long-term stability measurement was carried out. In this experiment, a photodiode with a small detection aperture was placed at the focal plane corresponding to the *f* = 48 mm state and used to monitor the focused optical power. The device was driven by a signal generator providing amplitude modulation between an OFF voltage of 10 Vpp and the ON voltage of 2.1 Vpp with a period of 60 s, so that the FZP was repeatedly switched between the non-focusing state and the 2*π*-wrapped focusing state. The measurement was run continuously for 24 h. In the subsequent analysis, only the data points corresponding to the ON state (2.1 Vpp) were retained in order to isolate the stability of the focusing behavior from the deliberate ON–OFF modulation.

The resulting power trace was measured (see Supplementary Information Fig. S5). The blue dots represent the raw power measurements for each ON interval, which exhibit short-term fluctuations arising from voltage changes from the OFF state to ON state, environmental perturbations (e.g., small temperature drifts or air turbulence), and detector/electronic noise. To reveal the underlying stability of the device, the data were further processed in two ways. First, a moving average (orange curve) was computed, which effectively suppresses the high-frequency noise and highlights slow drifts in the focusing efficiency. Second, a linear regression (red line) and a global mean (black dashed line) were extracted from the entire 24-h dataset.

The moving-average curve remains essentially flat over the full measurement window and closely follows the global mean, indicating that the average focusing power in the ON state does not degrade with time. The fitted linear trend exhibits only a very small slope, corresponding to a drift of less than 1% of the mean power over 24 h, which is within the expected level of laser and detector drift in our experimental arrangement. In other words, no systematic decay or fatigue of the electro-optic response is observed despite continuous cycling between 10 and 2.1 Vpp for more than 1.4 × 10^3^ switching events.

Furthermore, the response time of the device was examined by observing individual voltage cycling (see Supplementary Information Fig. S6). The rise time (defined as the change in power from 10 to 90% of its value) was somehow limited to 6.734 s, and the fall time was 0.245 s. The rise time is slow due to several reasons. Firstly, the FZPs were fabricated at high voltages (100 Vpp), where the LC is locked into a homotropically aligned state and thus the lens is only valid at 0 Vpp or low voltages. The rise time is controlled by switching the LC device from the high voltage state to a low voltage state, which is naturally slower than the opposite case. Secondly, this fabrication method resulted in a hybrid aligned nematic (HAN). Compared with a conventional homogeneous planar LC device, the HAN configuration introduces competing boundary conditions that impose a non-uniform hybrid director profile across the thickness. Consequently, the electro-optic response is governed not only by bulk Fréedericksz-like reorientation of the director but also by anchoring constrained profile reconstruction. These additional constraints can lead to slower apparent switching times, particularly in the relaxation process. Secondly, the thickness of this LC layer (20 µm) is too thick to be able to switch fast. Mathematically, the rise time in this case is related to the LC properties as follows:

3 $${\tau }_{rise}\approx \frac{{\gamma }_{1}{d}^{2}}{{\pi }^{2}K}$$

where *γ*_1_ is the rotational viscosity, *d* is the LC layer thickness, and *K* is the relevant Frank elastic constant. As can be realized from the equation, the rise time is proportional to the square of the LC layer thickness, which leads to such a slow response time. Comparably, the fall time is much quicker than the rise time, which is governed by:

4 $${\tau }_{fall}\approx \frac{{\gamma }_{1}{d}^{2}}{{\pi }^{2}K}\frac{1}{{\left(\frac{V}{{V}_{{th}}}\right)}^{2}-1}$$

where *V* is the applied voltage and *V*_th_ is the threshold voltage.

Overall, these results demonstrate that the polymer-stabilized phase profile and the LC director configuration are robust under repeated voltage changes. The device maintains a nearly constant focal intensity in its ON state over day-long operation, with only small fluctuations attributable primarily to the optical measurement system rather than to intrinsic degradation of the LC FZP. This long-term stability, together with reliable voltage-controlled switching between *f* = 24 mm and *f* = 48 mm, confirms that the demonstrated LC FZP is suitable for practical applications requiring sustained operation and repeated reconfiguration.

Building on this validated stability, future work will focus on improving both structural robustness and switching speed by optimizing the writing conditions and device architecture. To avoid the formation of a HAN-like configuration and to preserve a conventional planar nematic alignment at the desired voltages, we will perform laser writing at 0 Vpp. This approach is expected to improve both rise and fall times. However, as mentioned earlier, writing at zero bias voltage can lead to fuzzy and less stable polymer microstructures due to laser-induced thermal fluctuations and the increased susceptibility of the LC director to random motion at room temperature. To mitigate this limitation, we plan to implement active temperature control during laser fabrication (e.g., using a cooling stage) to stabilize the LC director and suppress thermally driven pattern blurring.

The current 20 µm LC layer thickness was selected to avoid unintended polymerization across the full layer due to the relatively long axial voxel in our present laser writing fabrication system. Employing higher NA objectives to reduce the *z*-voxel size should therefore enable thinner LC layers to be used with faster electro-optic response. Furthermore, for improved robustness and stable long-term use, replacing the photoinitiator IR819 with alternatives that are not susceptible to ambient light conditions, such as IR651, which has a shorter wavelength threshold for polymerization initiation, would lead to longer-term stability even in the presence of ambient white light illumination conditions.

## Discussion

In this study, we have developed a novel method for creating continuous phase FZPs within a nematic LC mixed with polymerizable reactive mesogens and photoinitiator. The approach involves using an Euler–Lagrange relaxation method to first derive the director profile necessary to achieve a wrapped FZP phase distribution, before TPP-DLW is then used to spatially vary the refractive index/phase profile through localized polymerization. Comparisons of simulated polarizing optical microscopy images with experimental observations confirmed the spatially varying phase profile within the LC device, which was also further corroborated using digital holographic microscopy.

The findings presented in this paper illustrate the viability of combining polymerizable nematic LCs with high-resolution laser writing to produce reconfigurable yet inherently efficient FZPs. The novel design of fabricating three-dimensional FZPs with higher degrees of phase wrapping provides extra functionality for the FZP, not only in terms of being able to turn ON and OFF the lens behavior, but also to be able to switch between different focal planes with the application of a voltage. Beyond potential applications in near-eye displays for AR/VR, such continuous phase FZPs may prove advantageous in other areas of photonics, including optical beam steering, compact imaging systems, and adaptive optics.

While the present devices are demonstrated at a millimeter scale, it is important to acknowledge that direct TPP-DLW that fabricating centimeter-scale monolithic apertures in a purely serial manner, remains a challenge, owing to the intrinsic slow voxel-by-voxel writing process and limited field of view. However, these constraints do not represent a fundamental obstacle to scalability. Through the use of parallelization (creating multiple voxels simultaneously using holographic beam splitting or interference-based exposure), it may be possible to significantly decrease the fabrication time and simultaneously increase the dimensions, leading to centimeter-scale fabricated optical elements. Such parallelization would enable the total functional aperture of tiled microlens arrays to readily reach the centimeter regime without relying on a single monolithic TPP-DLW write process^30,31^. Alternatively, the TPP-DLW adopted here could be utilized as a high-precision master fabrication platform, capable of generating a single continuous phase template with nanometric surface fidelity. This master can subsequently be transferred to a nickel shim via electroforming and replicated at wafer level throughput using UV nanoimprint lithography, providing a realistic route to large area, high volume production as reported elsewhere^32,33^. Together, nanoimprint replication and holographically parallelized microlens-array fabrication provide two practical and complementary pathways for scaling the proposed platform beyond the proof-of-concept devices shown here.

Future research may therefore extend the fabrication method to larger apertures as well as explore alternative LC formulations for broader spectral tunability or faster switching responses. Furthermore, by using higher birefringence LC materials with higher NA objectives in the laser writing process, 6*π* rad wrapping or even 8*π* rad wrapping could be achieved inside glass cells with 20 µm LC layer thicknesses, resulting in enhanced switchability of the focal length. Moreover, by stacking multiple LC layers with higher wrapping FZPs, the focal length could be switched over a large number of orders. In doing so, continuous-phase LC lenses could play a key role in the next generation of lightweight, power-efficient, and high-performance optical systems. By uniting the advantages of LC tunability with the high fidelity of TPP-DLW, this work highlights a promising route toward reconfigurable yet efficient diffractive flat optics suitable for next-generation photonic systems.

## Materials and methods

### Phase profile estimation

The unwrapped phase profile of the FZP can be expressed by

5 $$\phi \left(r\right)=\left(\frac{2\pi }{\lambda }\right)\left(\sqrt{{f}^{2}+{r}^{2}}-f\right)$$

where *ϕ*(*r*) is the phase, 2*π*/*λ* is the wave vector, *r* is the radial coordinate from the lens center, and *f* is the focal length. Figure 2a shows this unwrapped phase distribution, which reaches a maximum of 4*π* rad. As optical interference repeats every 2*π*, the phase was wrapped to remain within the 0–2*π* interval (Fig. 2b). This wrapping ensures the designed phase proϕile is effectively implemented while preserving the focusing properties of the lens.

For a varifocal switchable FZP which is designed to be wrapped into 4*π* rad, the first focus (*f*_1_ = *f*) is designed to work when the device is 4*π* rad wrapped, and the second focus (*f*_2_ = 2 *f*), where the phase is written as:

6 $$\frac{4k\pi }{\lambda }=\left(\frac{-2\pi }{\lambda }\right)\left(\sqrt{{f}^{2}+{r}^{2}}-f\right)$$

and

7 $$\frac{2k\pi }{\lambda }=\left(\frac{-2\pi }{\lambda }\right)\left(\sqrt{{\left(2f\right)}^{2}+{r}^{2}}-2f\right)$$

These two equations describe the ideal phase profile to achieve *f*_1_ = *f* in 4*π* rad wrapped phase and *f*_2_ = 2 *f* in 2*π* rad wrapped phase. By rearranging and simplifying, these equations become:

8 $$\frac{4k\pi }{\lambda }=\left(\frac{-2\pi }{\lambda }\right)\left(\sqrt{{f}^{2}+{r}^{2}}-f\right)\Rightarrow {r}_{4\pi }^{2}=\lambda \left(4\lambda -4f\right)$$

and

9 $$\frac{2k\pi }{\lambda }=\left(\frac{-2\pi }{\lambda }\right)\left(\sqrt{{\left(2f\right)}^{2}+{r}^{2}}-2f\right)\Rightarrow {r}_{2\pi }^{2}=\lambda \left(\lambda -4f\right)$$

As can be seen, the relationship between the *f* and *r*^2^ between a 4*π* rad wrapped phase with *f* = *f* and a 2*π* rad wrapped phase with *f* = 2 *f* is only ${{3}\lambda^{2}}$. This derivation indicates the small difference between their ideal phase profiles. On the one hand, this suggests that the varifocal switchable FZP lens can be practically achieved as the magnitude of the wavelength of light (nm) is far smaller than that of the radius of the FZP (mm). On the other hand, this derivation implies some imperfections in the far-field focusing, which might result in light leakage or a slightly different focal length compared to the design.

### Numerical simulation of the LC director profile

A one-dimensional model was employed to simulate the LC director profile in the region of interest for the continuous phase FZP. The cell was discretized along one spatial dimension *z*, where each layer was characterized by a director tilt angle *θ*(*z*). Under the single elastic constant approximation, the elastic and electrostatic energies were accounted for by solving the following Euler–Lagrange equation:

10 $$K\frac{{d}^{2}\theta }{d{z}^{2}}-{\varepsilon }_{0}\Delta \varepsilon {E}^{2}\sin \theta \cos \theta =0$$

where *K* is the elastic constant, *ε*_0_ is the dielectric permittivity of free space, Δ*ε* is the dielectric anisotropy, and *E* is the applied electric field. A small pre-tilt angle was used as an initial condition for each discretized layer, and a specified locked region was enforced at *θ* = *π*/2 to represent the polymerized layer introduced by TPP-DLW. A relaxation scheme was then applied, iteratively updating *θ* in each layer according to the local balance of elastic and electric torques, until convergence was achieved. After convergence, the resulting director profile was used to compute the optical phase difference Δ*φ* by integrating the effective refractive index *n*_eff_(*z*) through the cell thickness:

11 $$\Delta \phi ={\int }_{0}^{Z}\frac{2\pi }{\lambda }{n}_{eff}\left(z\right){dz}$$

where *n*_eff_(*z*) depends on the local tilt angle *θ*(*z*) and on the ordinary and extraordinary refractive indices. Repeating this procedure for various locked region thicknesses and different applied voltages yielded a tunable relationship between Δ*ϕ* and the polymerization height, as illustrated in Fig. 2c. Using the wrapped phase profile design, the necessary polymerization height distribution for TPP-DLW was then derived (Fig. 2d). Finally, the completed phase profile and corresponding height profile for the continuous phase FZP are shown in Fig. 2e, f, respectively.

### Numerical simulation of light propagation

Light propagation through the fabricated FZPs was modeled using scalar diffraction theory under the Fresnel approximation. A 2D fast Fourier transform (FFT) based propagation algorithm was implemented in MATLAB to efficiently compute the optical field at the focal plane. The simulation domain was defined as a square region with a physical width of *x* = 5 mm, sampled with 4096 × 4096 points, giving a spatial resolution of Δ*x* = *x*/*N*_*x*_. The illumination wavelength was set to *λ* = 632.8 nm, and the propagation distance was *z* = 7.5 mm, matching the designed focal length of the FZP. The output sampling followed the standard Fresnel scaling relation, *x*_out_ = *N*_*x*_*λz*/*x*. The FZP was modeled by a complex transmission function:

12 $${T}_{\mathrm{FZP}}\left(x,y\right)=\exp \left[-i\phi \left(x,y\right)\right]$$

where *ϕ*(*x,y*) is the phase profile of the lens. For a continuous phase FZP,

13 $${\phi }_{{\rm{cont}}}\left(x,y\right)=k\left[\sqrt{{f}^{2}+{x}^{2}{+y}^{2}}-f\right]$$

where *k* = 2*π*/*λ*, and for a binary FZP, the phase was quantized to two discrete levels, either 0 or *π*. The input field was assumed to be a circular top hat beam with uniform amplitude within the aperture. Propagation from the input to the output plane was computed using the Fourier transform form of the Fresnel diffraction integral^34^:

14 $$U\left({x}^{{\prime} },{y}^{{\prime} }{;z}\right)=\frac{{e}^{{ikz}}}{i\lambda z}{e}^{i\frac{k}{2z}\left({x}^{{\prime} 2}+{y}^{{\prime} 2}\right)}\times {\iint }_{-\infty }^{\infty }U\left(x,{y;}0\right){e}^{i\frac{k}{2z}\left({x}^{2}+{y}^{2}\right)}{e}^{-i\frac{2\pi }{\lambda z}\left({{xx}}^{{\prime} }+{{yy}}^{{\prime} }\right)}{dxdy}$$

where *U*(*x,y*;0) is the complex field at the input plane, and *U*(*x’,y’;z*) is the propagated field at a distance *z*. In practice, this equation was implemented using a two-step FFT algorithm: (1) multiply the input field by the quadratic phase factor ${e}^{i\frac{k}{2z}\left({x}^{2}+{y}^{2}\right)}$, (2) compute a 2D FFT, and (3) multiply by the output quadratic phase factor ${e}^{i\frac{k}{2z}\left({x}^{{\prime} 2}+{y}^{{\prime} 2}\right)}$. The intensity at the output plane was then calculated as

15 $$I\left({x}^{{\prime} },{y}^{{\prime} }\right)={{|U}\left({x}^{{\prime} },{y}^{{\prime} };z\right)|}^{2}$$

The resulting intensity maps were normalized and analyzed to extract focal spot profiles and peak intensities, enabling quantitative comparison between continuous and binary FZPs.

### Device preparation and fabrication

A glass cell (Instec) with an air gap of 20 μm with anti-parallel rubbed polyimide alignment layers and Indium Tin Oxide electrodes (area of 25 mm^2^), was used in this experiment. The polymerizable nematic LC mixture was prepared by mixing 78 wt.% of the nematic mixture E7, 20 wt.% of RM257 monomer (Synthon Chemicals Ltd), and 1 wt.% of the photoinitiator IR819. The relatively high concentration of RM257 was selected to ensure that the polymerized FZP structure forms a mechanically stable, field-insensitive polymer scaffold. Although RM257 reduces the effective birefringence of the mixture, it is itself anisotropic, and the resulting Δ*n* decrease remains modest. This reduction is fully accounted for in our simulations, where the effective Δ*n* was evaluated using a director-relaxation method based on the Euler–Lagrange equations incorporating the RM257 volume fraction and its refractive indices. The calculated Δ*n* is therefore sufficient to support the required 2*π*/4*π* phase modulation within the chosen cell thickness, consistent with the experimentally observed switching behavior. The 1 wt.% photoinitiator and polymer fraction do not impede alignment or electro-optic response in the unpolymerized regions, which exhibit stable switching. E7 was chosen as the host material due to its well-characterized properties and its favorable refractive-index compatibility with polymerized RM257, which contributes to good optical transmission.

The resultant mixture was then transferred to a hot plate at 70 °C. After capillary filling the LC mixture into the glass cell, it was then placed in the laser writing system where a Spectra-Physics Mai Tai Titanium-Sapphire laser (*λ* = 780 nm), delivering 100 fs pulses at a 80 MHz repetition rate, was incident on the device. The laser beam was focused using a 0.45 NA objective lens with 20× magnification. The LC glass cell was mounted on a three-dimensional translation stage, constructed by integrating an Aerotech ANT95XY 2D translation stage with an ANT95v vertical translation stage. During the exposure, a square wave voltage signal with a peak-to-peak voltage of 100 Vpp at a frequency of 1 kHz was applied to the cell. This signal was generated using a Tektronix AFG3021 signal generator and amplified by an FLC F10n AD amplifier.

The FZP was fabricated at a speed of 0.5 mm/s by the linear translational stage with an incident laser power of 40 mW. These parameters were selected to provide a sufficient polymerization dose for forming a mechanically stable and optically smooth polymer network while minimizing overexposure-related feature broadening and preserving the alignment of the surrounding unpolymerized LC. The same laser writing conditions were applied across multiple devices to ensure consistent fabrication of the 4*π* rad wrapped phase profile. The same parameters were used across multiple fabrication runs, and small deviations of the peak phase modulation were also realized due to sensitivities to cell thickness variance of the individual glass cells, as well as a slight tilt of the stage for each glass cell.

### Transmission measurements

The Transmission measurement of focus spots (see Supplementary Information Fig. S3) was developed for examining the focusing behavior of the continuous phase FZPs. A He–Ne CW Laser emitting at *λ* = 632.8 nm (shown in blue) was used as the light source. The LC FZP was mounted on the sample holder and connected to a combination of a signal generator and a voltage amplifier. The light then passed through an LP and an HWP for controlling the orientation of the linear polarization state of the laser. Then, a 4*f* optical system was used to adjust the laser beam size to fit the image onto a CCD camera. Then, the laser passed through the LC FZP, and a signal was received by the CCD camera. Another arm with an LED light source (shown in red) was reflected by the prism and also passed through the sample holder, and the signal was received by the CCD camera. This arm acted as a microscope, which was used to locate the position of the FZP in the LC cell relative to the incident laser beam.

Imaging measurements were performed to assess the imaging capability of the 4*π*-wrapped FZP. Illumination was provided by a 625 nm fiber-coupled LED (Thorlabs M625F2). The beam diameter was restricted using an aperture to match the 1.2 mm FZP aperture, ensuring consistent illumination across the active lens area. A USAF resolution target (Thorlabs R1L1S7N) served as the test object and was placed immediately after the aperture. The FZP was mounted on a sample holder at a defined object distance, and a CCD camera was positioned at the corresponding image plane to capture the transmitted images.

### Polarized optical microscope images

Polarizing optical microscope (POM) images of the continuous phase FZP were captured by using an Olympus BX51 polarizing optical microscope, in conjunction with a QImaging R6 Retiga camera and an Olympus LMPLFLN5× objective lens. A Thorlabs FB660-10 narrowband filter was placed in the illumination path to prevent polymerization by the microscope light source post fabrication. The devices were driven by a Multicomp MP750510 AC square wave signal generator operating at 1 kHz with high impedance (High Z), and images were acquired for peak-to-peak voltages ranging from 0 to 10 Vpp. POM measurements were performed with the signal generator in the High-Z configuration, ensuring the full programmed voltage was applied; in this low voltage regime, the choice of output impedance has only a negligible effect on the LC switching behavior.

### Digital holographic microscope images

The 3D phase map was captured by a digital holographic microscope. The main structure of this microscope system is a Mach–Zehnder (M–Z) interferometer. A 2 mW He–Ne laser (Thorlabs HRP020-1) was focused by a 150 mm lens onto the LC FZP device, and an objective lens (Olympus UPlanFL N 10× NA:0.3 Objective) was used to collect the transmitted signal of the sample arm. In the M–Z interferometer, the sample arm and the reference arm have the same type of imaging system, with the same objective lens and 150 mm tube lens to ensure a match of the wavefront. The second beam splitter can tune its rotation and tilt to make the ±1st orders separate from the central 0th order. A high-resolution camera (Teledyne Retiga E7 CMOS Camera) was used to capture the hologram after the second beam splitter. After the Fourier transform of the hologram, the separated 1st order was manually selected, and an inverse Fourier transform was applied to reconstruct the scalar complex field. A fast 2D phase unwrapping algorithm was used to calculate the 3D phase map from the wrapped phase signal contained in the reconstructed scalar field.

## Supplementary information

Supplementary Information

## Data Availability

The authors declare that all data supporting the findings of this study are available within the paper.
