# Supplementary material for: Electrically switchable continuous phase liquid crystal Fresnel zone plate
Source: Light Sci Appl. 2026 Apr 17;15:203. doi: 10.1038/s41377-026-02251-3 (PMC13087270; doi:10.1038/s41377-026-02251-3)
Supplement: Supplementary file 1 — Supplementary Information [file 41377_2026_2251_MOESM1_ESM.pdf]

Supplementary Information

Electrically Switchable Continuous Phase Liquid Crystal Fresnel Zone Plate

Zhiyu Xu*, Camron Nourshargh, Tianxin Wang, Alec Xu, Nathan Spiller, Urban Mur, Martin J. Booth, Steve J. Elston, and Stephen M. Morris*

Department of Engineering Science, University of Oxford, Oxford, OX1 3PJ, United Kingdom

Correspondence: zhiyu.xu@wolfson.ox.ac.uk or stephen.morris@eng.ox.ac.uk

Keywords: Liquid crystals, Fresnel Zone Plates, two-photon polymerization, direct laser writing

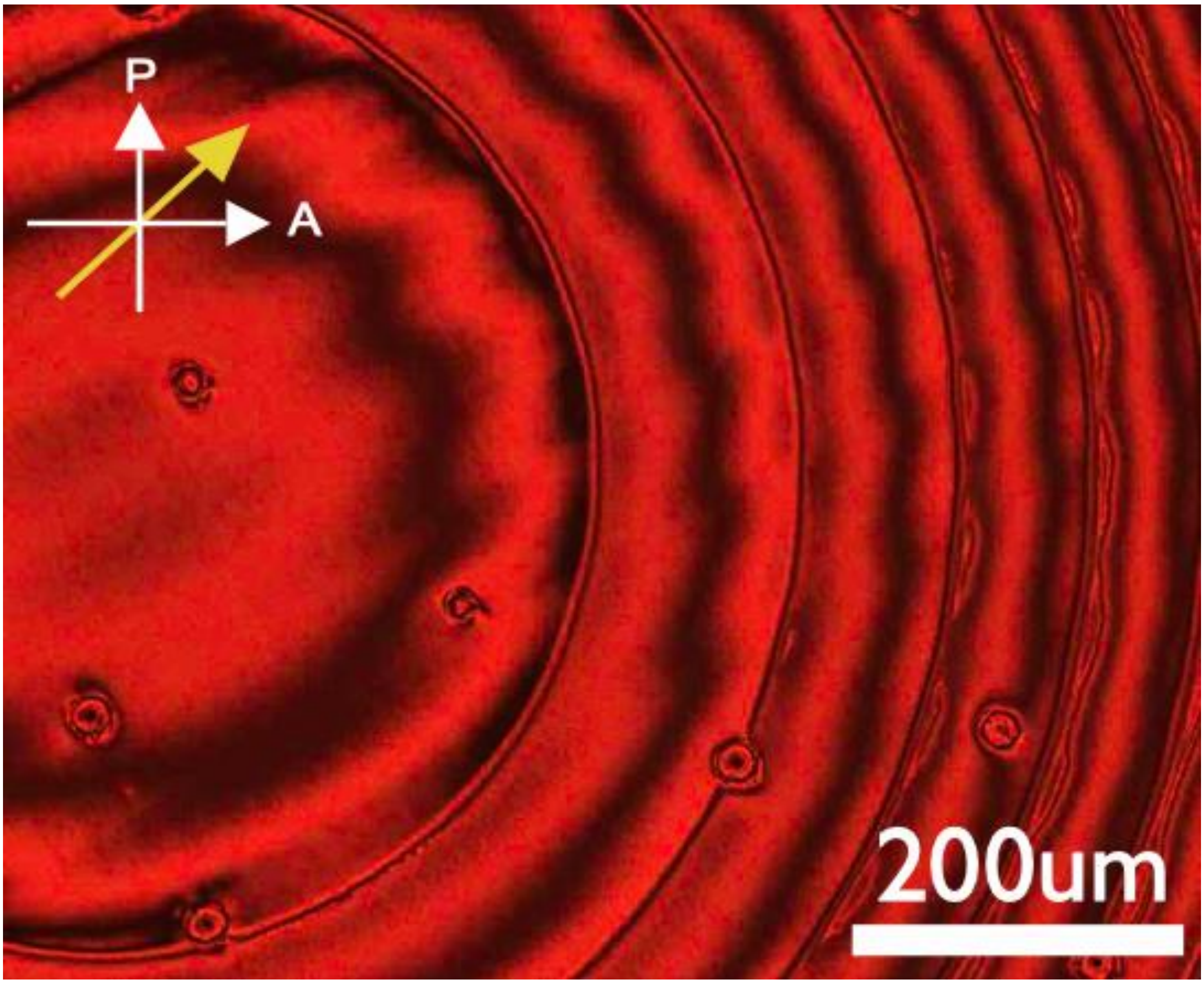

Figure S1. Polarising optical microscope (POM) image of a continuous phase Fresnel Zone Plate fabricated using a lower concentration of the reactive mesogen RM257 in the polymerizable liquid crystal (LC) mixture filled into a glass cell with a gap of 20 μm. The polymerizable LC mixture was composed of 15 wt.% RM257, 1 wt.% IR819 and 84 wt.% E7. The single-headed white arrows represent the directions of the polariser (P) and analyzer (A), and the single-headed yellow arrow represents the orientation of the rubbing direction of the alignment layers. These measurements were obtained at a temperature of 25 °C

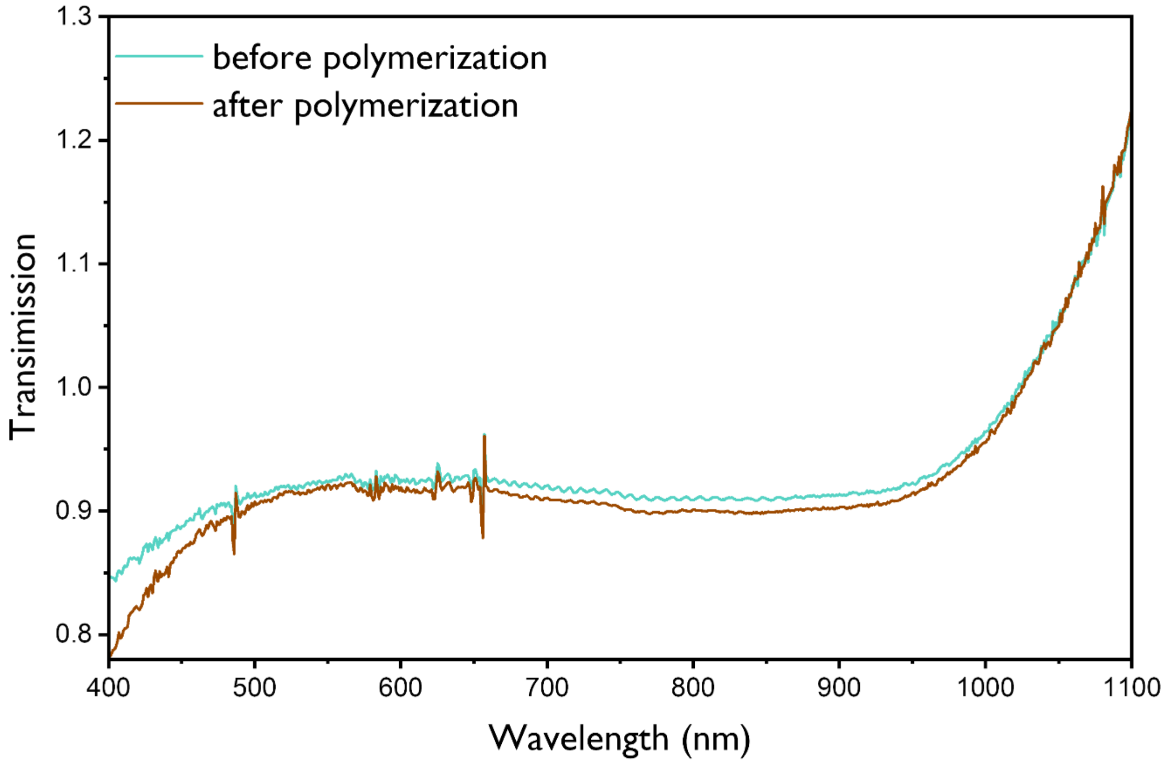

Figure S2. The transmission as a function of wavelength for the polymerizable nematic liquid crystal mixture (15 wt.% RM257, 1wt.% IR819 and 84 wt.% E7) filled into a glass cell with a gap of 20 µm before (light green line) and after (red line) polymerization. These measurements were obtained at a temperature of 20 °C

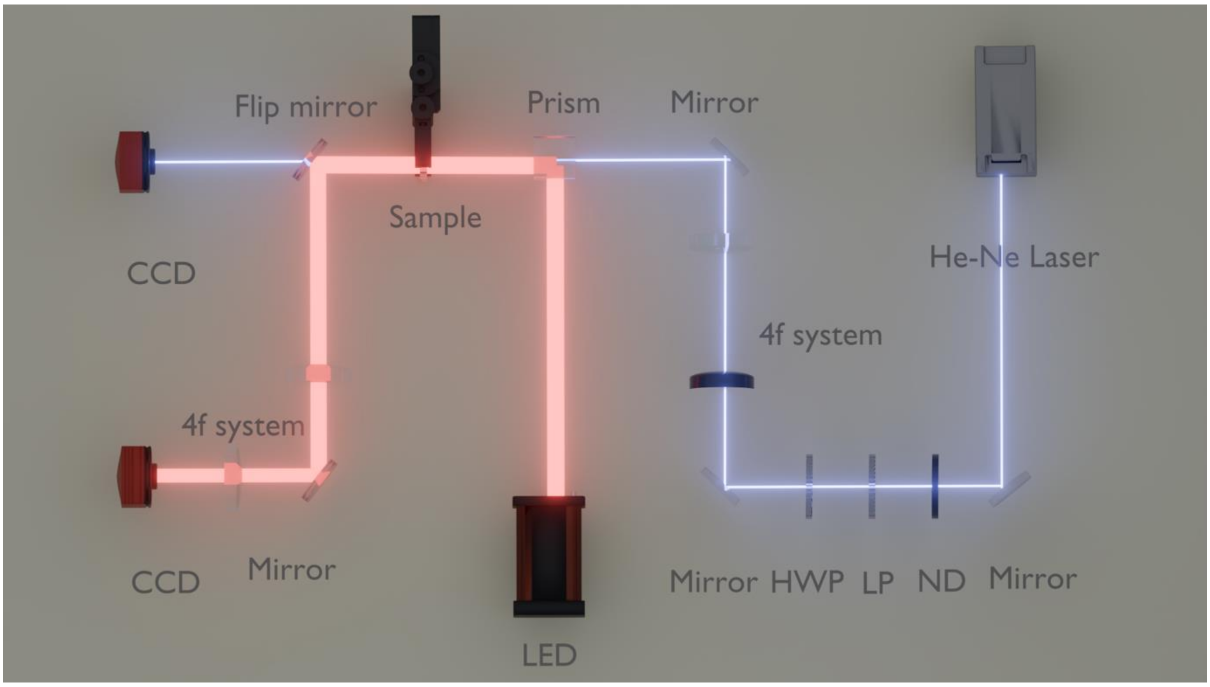

Figure S3. Experimental configuration for measuring the focussing properties of the laser-written nematic liquid crystal (LC) Fresnel Zone Plate (FZP). A linear polarizer (LP) and a half-wave plate (HWP) were used to control the polarization of the incident laser source at the LC FZP. ND is a neutral density filter.

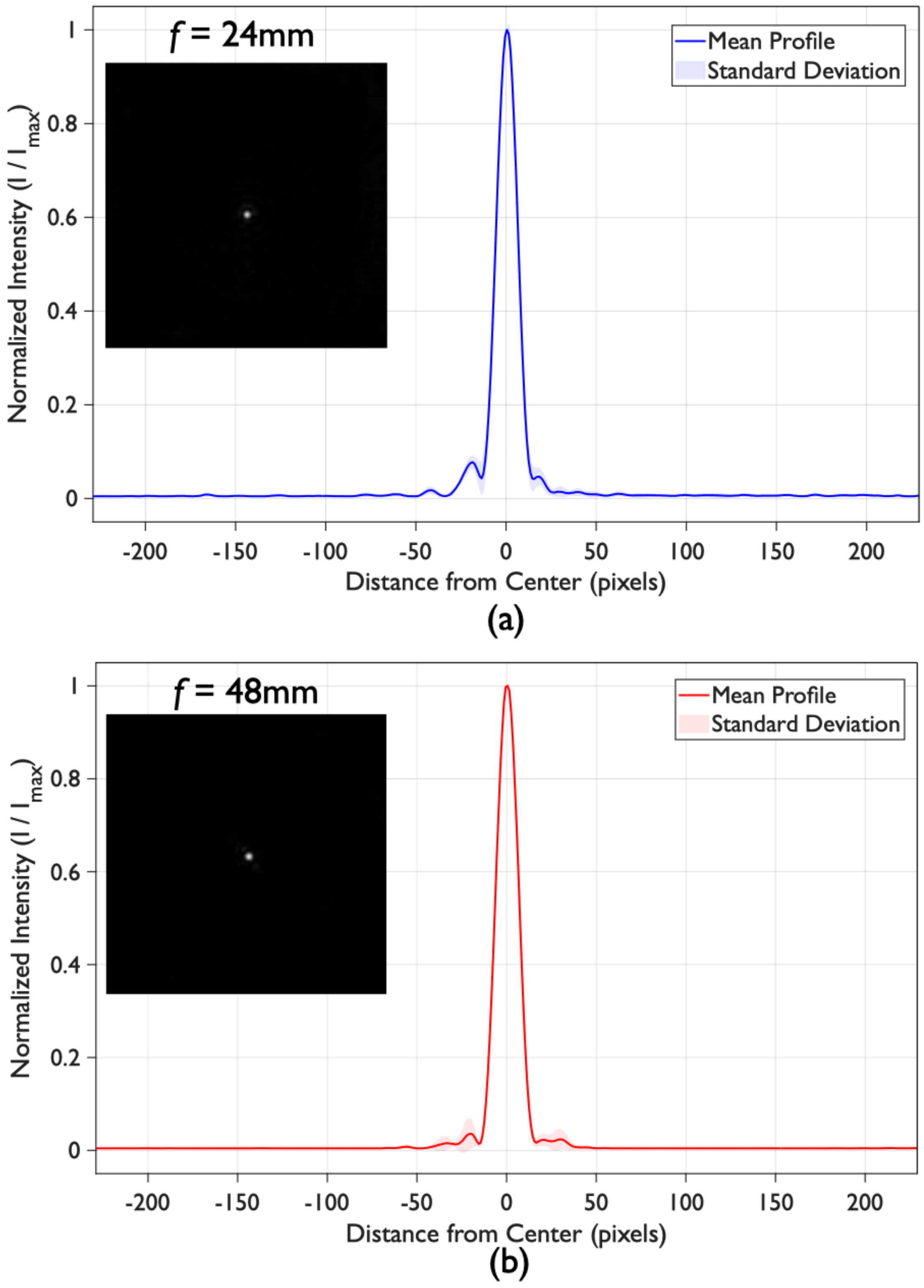

Figure S4. The intensity profile of the experimentally measured focus spot at (a) f = 24 mm and (b) f = 48 mm. An azimuthally averaged radial intensity profile was obtained by rotating a line passing through the focal-spot centre and averaging the corresponding line-scan intensities across all angles. The inset images are experimentally obtained images of the focal spot at the corresponding focal length. These measurements were obtained at a temperature of 20 °C.

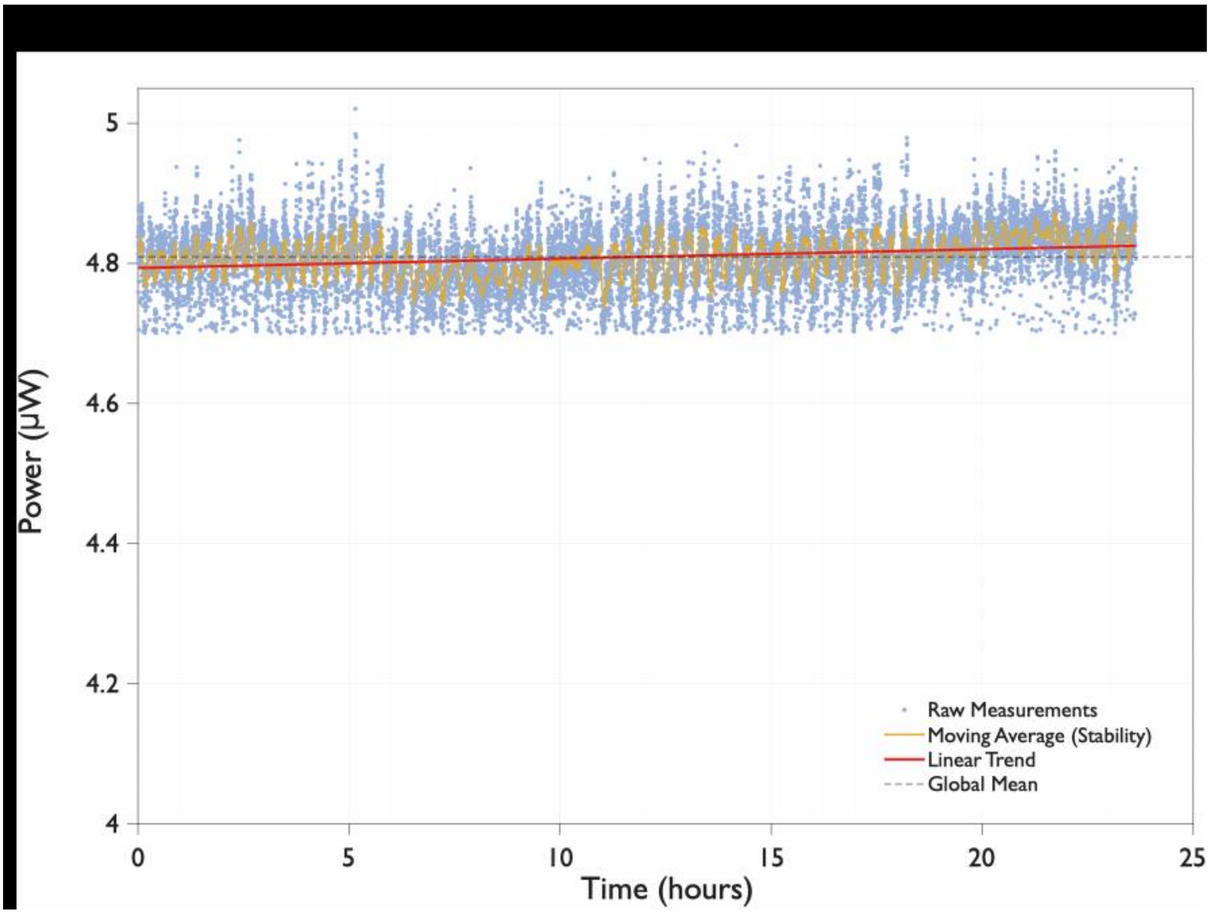

Figure S5. Stability test of the continuous phase 4π wrapped Fresnel Zone Plate (FZP). The results show the power recorded at a photodiode positioned at the focal plane as a function of time. A collimated laser was incident on the LC FZP and an aperture was used to only allow the focused beam from the FZP to pass through to a photodiode positioned at the corresponding focal plane. The device was connected to a function generator that was set to an amplitude modulation mode whereby the voltage changed every 60s, switching between 2.1 V and 10 V in order to switch ON the FZP with a focal length of $f = 48$ mm and then switching OFF. The measurements were obtained for the ON states only to indicate the stability of the FZP after switching. These measurements were obtained at a temperature of 20 °C.

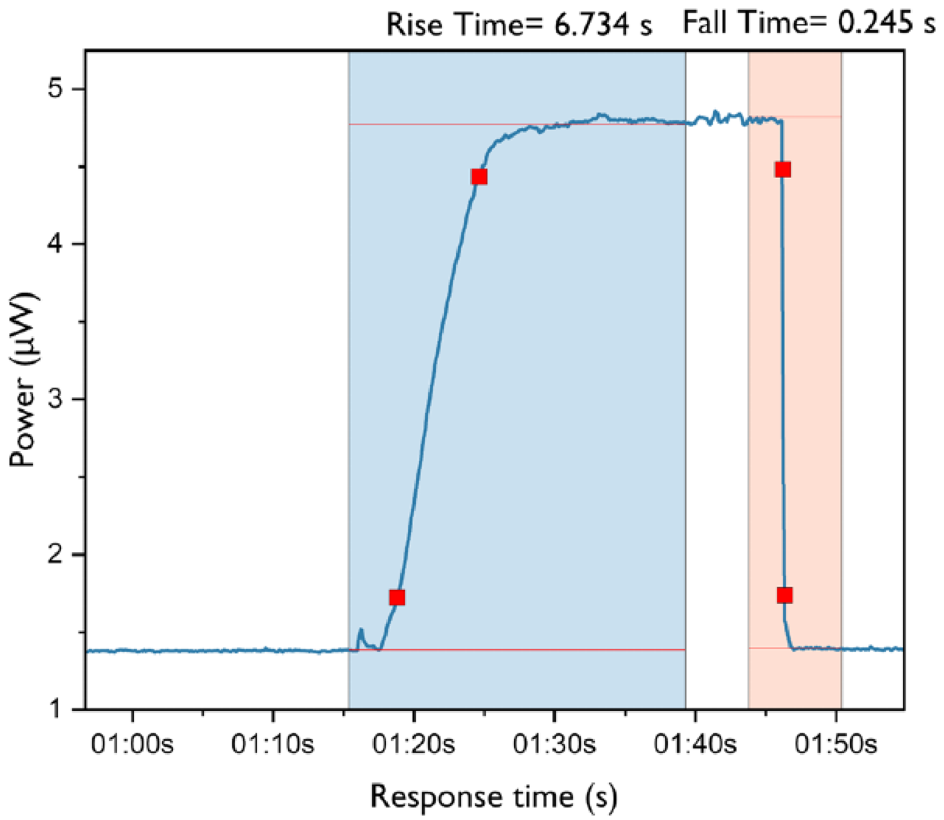

Figure S6. Measurements of the power recorded at the focal plane as a function of time, highlighting the rise time (change in power from 10% to 90% of the value) and the fall time (change from 90% to 10%) when switching ON/OFF the continuous phase Fresnel Zone Plate (FZP). The LC cell was filled with polymerizable LC mixture (20 wt.% RM257, 1wt.% IR819 and 79 wt.% E7). A photodiode with a small aperture was used to measure the focusing power of the FZP from an incident laser diode with a central wavelength of λ = 632 nm. These measurements were obtained at a temperature of 20 °C.
